# Psoriasis

**Published:** 1906-04-07

**Authors:** 


### PSORIASIS.

Dr. T. Robinson [1] states that next to eczema psoriasis is the commonest of skin diseases. Some 90 per cent. of all the remaining cases of skin disease are of this variety. He considers that although in a family one member of which is affected with psoriasis it is usual to find others presenting skin lesions, yet it is rare to find two instances of psoriasis in the same family.

The essential lesion of the disease is a papule, scaly from the first. Although large tracts of the skin may show the lesions of this disease, it is never universal: nor does the lesion of psoriasis ever leave either scar or pigmentation. A certain degree of itching usually attends the malady, but not always. Although the elbows and knees are the seat of election for the scaly patches of psoriasis, it is not true to say that the evidences of disease are always more marked in these situations than elsewhere; for even in well-marked instances it may be impossible to detect the least sign of the ailment in these favourite positions. Psoriasis is common on the scalp, and always affects the hair to some extent, either in the direction of depilation or loss of lustre. Sufferers from psoriasis are liable to a curious condition of the nails. This condition does not affect all the nails, neither is it symmetrical. The diseased nails are brittle and coarse on the surface. It is common to find beneath the nail a patch of psoriasis which permits the insertion of a probe between the nail and its bed without the causation of pain.

The causation of the disease is unknown. Unna strongly suspected a parasitic origin, but there is no evidence that psoriasis is capable of direct transmission. The diagnosis has to be made from pityriasis rosa, seborrhœa, and syphilis. The first is practically limited to the trunk and is but slightly scaly: it may be very difficult to distinguish psoriasis from seborrhœa, but the latter is a more acute disease and more amenable to treatment. Scaly syphilides are very like psoriasis, but seldom reach the size of the patches in the latter disease. The syphilitic patches do not commonly exceed half an inch in diameter. As regards the treatment the author states that the truest and frankest expression of the facts is to say: "We can promise to get rid of the eruption if you will persevere with the treatment; but we must warn you that in all probability your eruption will reappear at some future time." Of internal remedies the best are arsenic, salicylate of soda, and thyroid extract. In long-standing cases a pill containing one-twentieth of a grain of arsenic (*sic*), one grain of black pepper, and one grain of sulphate of iron may be given three times a day. In recent cases five-grain doses of salicylate of soda appear to be efficacious. In the case of women at the menopause thyroid extract appears to exert a very beneficial effect. For local treatment, first the scales must be removed, best by wet-packing, or failing that by means of a hot bath combined with a free use of soap. Subsequently the spots should be treated with an ointment composed of the tar, ammoniated mercury, and salicylic acid ointments in equal proportions.

[1] Med. Press, March 21, 1906.

